# The problem with unadjusted multiple and sequential statistical testing

**DOI:** 10.1038/s41467-019-09941-0

**Published:** 2019-04-23

**Authors:** Casper Albers

**Affiliations:** University of Groningen, Groningen, Netherlands

**Keywords:** Statistics, Research data

## Abstract

In research studies, the need for additional samples to obtain sufficient statistical power has often to be balanced with the experimental costs. One approach to this end is to sequentially collect data until you have sufficient measurements, e.g., when the *p*-value drops below 0.05. I outline that this approach is common, yet that unadjusted sequential sampling leads to severe statistical issues, such as an inflated rate of false positive findings. As a consequence, the results of such studies are untrustworthy. I identify the statistical methods that can be implemented in order to account for sequential sampling.

In experiments, researchers must balance between two competing arguments with respect to the sample size. On one hand, the sample size must be large enough to have sufficient power for accurate statistical inference. On the other hand, each additional observation comes at a cost and, especially when performing medical experiments or working with test animals, the researcher has the ethical obligation to avoid unnecessary oversampling.

A seemingly appealing approach is to sequentially collect data, one measurement at a time, and stop when you have sufficient measurements, e.g. when the *p*-value drops below 0.05. However, this approach also invalidates the statistical tests and biases the estimates, which is why it is usually labeled as a questionable research practice^1^. Quite often the description of the data collection in a paper is insufficient to check whether this approach has been followed or not. This is peculiar, because explicitly stating how the sample size was decided upon is advised by many academic associations, such as the Animal research association NC3Rs (item 10b in the ARRIVE guidelines^2^) and the American Psychological Association APA^3^. Furthermore, in the field of animal research, researchers usually must “assure an ethics committee that the proposed number of animals is the minimum necessary to achieve a scientific goal”^4^.

In various anonymous large-scale surveys, large numbers of researchers, active in various fields of research, have admitted to following this strategy at least once. Some of the findings include 36.9% of ecologists and 50.7% of evolutionary biologists^5^. For psychologists, the estimates include 55.9% for American^1^, 53.2% for Italian^6^, and 45% for German^7^ psychologists. Thus, the issue is widespread and occurs in a variety of scientific fields.

The problem with multiple statistical testing is more often recognized in the context of multiple independent testing. In this scenario, due to a large number of statistical tests being performed, the number of false-positives is increased and this needs to be corrected for (Fig. 2). Corrections such as the Bonferroni-correction are included in most statistical textbooks. If the null hypothesis holds true, a single statistical test will yield a false positive, so *p* < 0.05, in 5% of the times. This 5% is something many scientists think is an acceptably small probability for incorrectly rejecting the null hypothesis (although you can make a motivated choice for another rate^8,9^). When, for instance, performing 10 independent tests, whilst H_0_ is true, then the probability of finding at least one false positive is equal to 1 – (1 − 0.05)^10^ = 40.13%, very high. The Bonferroni-correction, and other corrections, ensure that this so-called familywise error rate remains at an acceptable level.

As most editors and reviewers are aware of the need for multiple testing, it rarely happens in published research that authors explicitly abstain from any correction for multiple testing. This does not imply that this practice is without problems. First, it is not straightforward to decide which tests within a single paper constitute the ‘family’ for which the familywise error rate needs to be capped at 5%^10,11^. Consider, for instance, the common situation of a two-way ANOVA. Here, one performs three tests: a main effect of each of both ‘ways’ plus an interaction. Yet, researchers rarely correct for this^12^.

Second, correcting for many tests has a deteriorating effect on the statistical power (too often not rejecting H_0_ even though it is false^13^). Third, one could present fewer comparisons than were actually performed, and thus employ a more lenient correction. For instance, when a study has been performed where three groups were mutually compared, the Bonferroni-adjusted *α*-level would be 0.05/3 = 0.0167. By omitting one group from the paper, the *α*-level for the comparison between the remaining groups could remain at 0.05. This research practice is clearly questionable, yet not uncommon^1^.

Things are different, and much less well-known for sequential testing. Sequentially collecting data until some threshold is reached doesn’t have to be problematic, as long as you employ an appropriate correction. Here, I outline the problem and indicate what can be done to deal with this. I will demonstrate this based on the well-known *t*-test as the simplicity of this test works for demonstrative purposes. The issue is not exclusive to the *t*-test, and holds for all significance testing procedures.

Suppose you want to perform an independent samples *t*-test. You begin with *n* = 2 measurements per group (with 1 measurement per group you cannot compute the within-group-variance, and thus cannot conduct a *t*-test). You perform the experiment, take your measurements and conduct your *t*-test. If *p* < 0.05, you stop collecting more data, else you collect one more measurement per group. Again, you conduct the analyses and conduct the *t*-test. This approach continues until you either have *p* < 0.05 or have run out of resources to collect more data or reached a pre-decided stopping point.

When performing independent tests, the FDR for *k* tests can be computed via the formula 1 – 0.95^*k*^. When doing sequential comparisons, the situation is somewhat different: the subsequent tests are not independent, as they are partly based on the same observations. For instance, the *p*-value for the test after 25 measurements is largely based on the 24 observations that were the basis of the previous *p*-value. Still, the multiple testing issue remains—albeit not as severe as with independent tests. It is possible to prove mathematically^14^ that with such a sequential approach it actually is guaranteed that at some point, the *p* value drops below 0.05, and also that at some later point, it again is above this threshold when H_0_ is true.

An example is given by the thick line Fig. 1. This figure is based on a computer simulation in the situation that H_0_ is true: there is no effect—both groups are not different and claiming a significant result constitutes a false discovery. The sequential approach outlined above has it’s first significant result for *n* = 42. Stopping the data collection here would enable the researcher to write a paper with a significant effect. However, for *n* = 43, the *p*-value would not be significant anymore. It crosses back and forth over the significance threshold a couple of times before the end of the plot. At *n* = 150, we’re kind of back where we started, with a very non-significant *p* value.

**Fig. 1:**
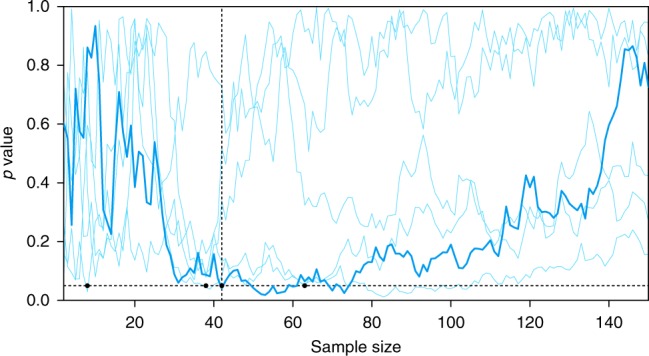
A computer simulation of sequential *p*-values when there is no effect. The thick line is the instance discussed in the text; the five thin lines represent independent simulations. The black dots indicate the first instance where one of the runs falls below the 0.05 level. Two of the runs don’t reach 0.05 before *n* = 150

This is of course just a single simulation. With other randomly generated data, the pattern will be different, as can be seen by the thin lines in Fig. 1. Note that for different trials of the simulation, the value dips below 0.05 at different number of trials (black dots in Fig. 1). To study how severe the problem is, I simulated 10,000 of these sequential strategies, and recorded at what sample size significance was reached for the first time. Figure 2 displays the results of this simulation.

**Fig. 2:**
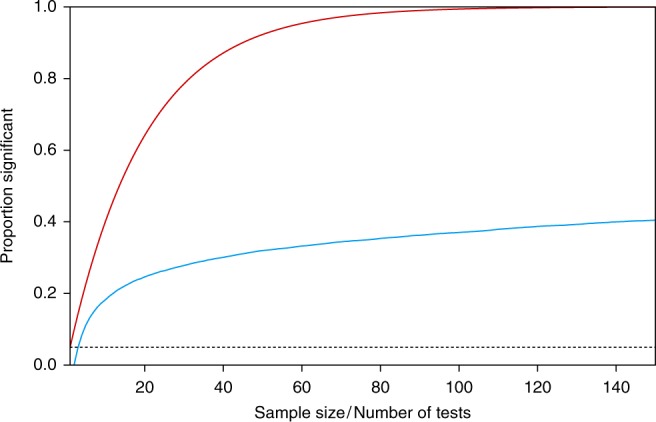
False discovery rate for unadjusted sequential testing (blue curve) and uncorrected multiple independent testing (red curve)

As can be seen, the issue is very severe—although less severe than the case of uncorrected multiple independent tests. Even if you would apply some rule where you stop collecting new data once *n* exceeds, say, 25, your false discovery rate exceeds 25%. Rather than the one-in-twenty chance of labelling a null result significant, we have a one-in-four chance, five times higher than intended.

Note that this problem not only affects the *p*-values, but also the estimates themselves. With sequential sampling, with each step the distance between the means of both groups will sometimes increase, sometimes decrease—simply due to coincidence. If we continue sampling until the means of both groups are sufficiently far apart in order to call it significant, we overestimate the effects. Thus, not only is the significance biased, so is the effect size.

So, in an attempt to require as few measurements—whether it concerns animals, participants, or something else—as possible for the experiment, this strategy would actually invalidate a study. Even more worrisome, it does so in a way that cannot be corrected for in a later stage. Thus, the informational value of the study is diminished, such that a new study is needed. In the end, this leads to more test animals/participants/etc. being needed, rather than less.

I outlined why unadjusted sequential testing is problematic. (Note that I’m by far not the first to do this, see e.g.^1,15^ and the references therein.) This does not imply, however, that the concept of sequential analysis—increasing your sample size in small bits until you meet some threshold—is not a good idea. It actually is a good idea, provided the necessary corrections have been made, as it safeguard against taking a sample larger than necessary (ref. ^16^, p.448,449). There are two classes of such sequential approaches: interim analyses (also known as group sequential analyses) and full sequential analyses.

In interim analysis^17,18^ one pre-specifies when one wants to inspect the data, e.g. both halfway at *n*_1_ = 50 and after collecting *n*_2_ = 100 measurements. If one tests with *α* = 0.029 at *n*_1_, and stops when the result is significant or to continues until *n*_2_ and tests again at this *α*-level, then the overall FDR is equal to 0.05. An advantage to non-sequential testing is that in case of sufficient evidence, one can stop data collection halfway through the process.

In full sequential approaches, one doesn’t check the data at a few pre-specified points, but after every observation. Theories about this by Abraham Wald^14^ and Alan Turing^19,20^ date back to the 1940s. These sequential approaches are more technical than standard methods. Wald’s procedure, for instance, involves computing the cumulative log-likelihood ratio after each observation, and stopping when this sum leaves a pre-specified interval (*a*, *b*). The computation of this log-likelihood ratio is far from straightforward. Statistically, this is the optimal approach of deciding upon the sample size. In interim analysis, one can stop data collection early in case there is sufficient evidence to reject H_0_. This is the same with the full sequential method, but here one can also stop when it is sufficiently clear that H_0_ will not be rejected. In practice, however, it is not always feasible to employ this approach, for instance when participants need to undergo group therapy in groups of size 20. In such contexts, interim analysis is an appealing alternative.

For sequential testing, much less (easy-to-use) software is available as for more conventional methods. Overviews of are available^21^. Apart from specifically programmed software and packages for R, which are not always straightforward for the practical researcher, interim testing is also possible in the statistical program SAS (ref. ^22^, (Chapter 109)). So far, for the full sequential method, it seems that the applied researcher cannot rely on easy-to-use software, the few R packages that deal with this method lack tutorials. One has to work through extensive technical textbooks^23,24^ in order to use this method, which explains why this method is so little used in practice, with the exception of the field of industrial statistics. Fortunately, employing the interim approach, instead of the conventional method of deciding upon the sample size based on a power analysis, can already provide large benefits. If researcher would employ this method more, precious resources would be saved.

For years, researchers interested in sequential methods were told to seek professional statistical help (ref. ^16^, p.455). It wasn’t until recently that attempts have been made to make the matter of sequential and, specifically, interim testing more accessible to researchers in other fields. In Table 1 the various approaches are summarized, with references to further reading. Hopefully, such efforts make this methodology more accessible to non-statisticians.

**Table 1:** Overview of different approaches towards sequential testing

Approach	Description, advantage and disadvantage	Mathematical complexity	Sample size required	Further reading
Non-sequential analysis	Collect a single sample, perform the analyses afterwards. Advantage: straightforward approach. Disadvantage: one might collect much more data then was necessary	Low	Largest	This is the classical approach, to be found in most statistical textbooks.
Interim analysis	A priori specify how often and when you analyze the data so far. At each point, test at adjusted alpha-level and stop when significant. Advantage: no specialized software required, in principle. Disadvantage: not as efficient as the full sequential approach.	Medium	Medium	ref. ^17,21,25,26^
Full sequential analysis	No a priori specifications required. Compute a certain, sample-based, statistic after each observation and stop collecting new data when it falls outside certain limits. Advantage: optimal w.r.t. deciding sample size. Disadvantage: specialized software required. Not always feasible.	High	Smallest	ref. ^23,24,27^

The described levels of mathematical complexity and sample size are relative to the other approaches. The indicated sample size is on average: for each approach it is possible by chance that it leads to a considerably larger or smaller sample
